# Probiotics Bring New Hope for Atherosclerosis Prevention and Treatment

**DOI:** 10.1155/2022/3900835

**Published:** 2022-09-24

**Authors:** Taiyu Zhai, Pingping Wang, Xiumei Hu, Lei Zheng

**Affiliations:** Department of Laboratory Medicine, Nanfang Hospital, Southern Medical University, Guangzhou, Guangdong 510515, China

## Abstract

Cardiovascular disease is the leading cause of human mortality and morbidity worldwide. Atherosclerosis (AS) is the underlying pathological responsible in most acute and severe cardiovascular diseases including myocardial infarction and stroke. However, current drugs applied to the treatment of AS are not clinically effective, and there is a large residual risk of cardiovascular disease and multiple side effects. Increasing evidence supports a close relationship between microorganisms and the incidence of AS. Recent data have shown that probiotics can improve multiple key factors involved in the development and progression of AS, including cholesterol metabolism imbalance, endothelial dysfunction, proinflammatory factor production, macrophage polarization, intestinal flora disturbance, and infection with pathogenic microorganisms, and therefore probiotics have attracted great interest as a novel potential “medicine”. This review is aimed at summarizing the effects of probiotics on various influencing factors, and providing valuable insights in the search for early prevention and potential therapeutic strategies for AS.

## 1. Introduction

Atherosclerosis (AS) is a chronic inflammatory disease caused by metabolic disorders, which has been recognized by most experts in this field. Persistent diseases such as hypertension and hypercholesterolemia or disorders of vascular bifurcation result in damage to the vascular endothelium, in which low density lipoprotein (LDL) undergo massive deposition and subsequently oxidized to ox-LDL, which causes inflammatory response and dysfunction of vascular endothelium, induces macrophage to foam cell transformation, enhances inflammatory response and promotes atherosclerotic plaque formation [1, 2]. Therefore, the progression of AS strongly depends on factors such as dyslipidemia, hypertension, and inflammatory response. However, therapeutic drugs developed for these progressive factors do not achieve satisfactory results and carry a huge residual risk of cardiovascular disease as well as various side effects [3–5]. It is suggested that there may be other influencing factors that mediate the progression of AS and prompted the search for new means of atherosclerosis prevention and treatment.

Recently, it has been found that microorganisms may also be important influencing factors during the progression of AS. There is increasing evidence that alterations in the composition of the gut microbiota and its metabolic potential have been identified as contributing factors in the development of AS [6, 7]. In a study of Chinese AS patients, the gut microbiota with atherosclerotic cardiovascular disease was analyzed by 16S sequencing, and it was found that the microbial composition in the gut of patients with coronary atherosclerosis was significantly different from healthy volunteers and that the compositional differences in gut microbes could be used for risk prediction of AS [8]. Apart from this, many kinds of metabolites produced by microorganisms are key substances for the interaction between microorganisms and hosts that can be directly derived from the transformation of bacteria, diet or host-derived substrates, and play an important role in the pathogenesis of metabolic disorder diseases [9–11]. For example, microbes are associated Trimethylamine N-Oxide (TMAO) and short-chain fatty acids (SCFAs), which have all been shown to be associated with the progression of AS [12, 13]. Therefore, atherosclerotic plaque formation may be influenced by microbes distally or directly in the vessel wall, and the detection of bacterial DNA components in atherosclerotic patient plaques provides strong support for this notion [14, 15].

Among the numerous microbial components detected in plaques, except for pathogenic bacteria such as Helicobacter pylori, the emergence of probiotics has provided new hope for the prevention and treatment of AS. In the study of Mitra et al., the nucleic acid component of probiotic Lactobacillus rhamnosus was detected in atherosclerotic plaques and was greatly enriched in stable plaques, suggesting that Lactobacillus rhamnosus may have an important role in regulating plaque stability [16]. Although the specific mechanism by which the nucleic acid components of probiotics are enriched in plaques is not clear, the role of probiotics in AS still attracts extensive attention. Probiotics have beneficial effects on host health when given in appropriate amounts [17].

Common probiotics mainly include Lactobacillus, Bifidobacterium, Lactococcus, Streptococcus, and Enterococcus, which can regulate many physiological activities in the human body, including digestion, metabolism, immunity, and competitive exclusion of pathogens. In addition to producing a variety of metabolites, including vitamins, SCFAs, and other substances, probiotics also play an important role in the conversion of nutrients and the prevention and treatment of numerous diseases [18–20]. Probiotics have achieved some success in preventing and treating diseases such as inflammatory bowel disease, diabetes, and cancer [21–23]. For AS, probiotics may directly or indirectly delay the progression of AS through the following mechanisms: regulating lipid metabolism, improving vascular endothelial function, and affecting macrophage polarization in vivo [24–26]. In addition, probiotics have been shown to affect some novel influencing factors associated with AS, including gut flora dysbiosis and pathogenic bacterial infection. Studies have shown that appropriate supplementation with probiotics or their associated products is able to improve the function and composition of the intestinal flora, combat pathogenic bacteria, and adjust the inflammatory status in the body [27, 28]. In this review, we will provide an analysis of the role of probiotics in AS and the prospects of possible clinical applications, exploring novel clinical diagnostic, and therapeutic strategies for AS and atherosclerotic cardiovascular disease (ASCVD).

## 2. Effects of Probiotics on Traditional AS Progression Factors

AS is a chronic inflammatory disease of the vascular system with a complex pathogenesis and slow disease progression and many factors can affect the progression of AS, including dyslipidemia, hypertension, inflammatory factors, smoking, obesity, age, and genetic factors. A number of reviews have provided a detailed analysis of the pathogenesis of AS, with disorders of lipid metabolism, endothelial dysfunction, inflammatory factors, and macrophages regarded as major drivers of atherosclerotic plaque formation and progression [29, 30]. Along these lines, many recent studies have highlighted the promising applications of probiotics.

### 2.1. Cholesterol Level Reduction by Probiotics

The occurrence and development of AS are affected by many factors, and disturbed lipid metabolism is a fundamental feature of most patients with AS, in which numerous epidemiological studies on low-density lipoprotein cholesterol (LDL-C) and total cholesterol (TC) have shown that they promote the occurrence and development of AS and have a guiding role in the clinical diagnosis of AS or ASCVD [31–34]. Regulating cholesterol levels in the body is therefore an important means of preventing the progression of AS. However, many clinical trials of antiatherosclerotic drugs have not yielded good results, and they have considerable residual risk of cardiovascular disease and various side effects [35–37]. Excitingly, recent studies have shown that multiple probiotics have beneficial regulatory effects on lipid metabolism in humans, thereby helping to delay the progression of AS or ASCVD.

Several Lactobacillus strains have been reported to display potent hypocholesterolaemic activity in vivo and in vitro. In C57BL/6 male mice fed a high-fat diet, after dietary intervention with Lactobacillus rhamnosus JL1 (1×10^9^ CFU/mL), the TC, TG, and LDL-C contents were significantly decreased, and the HDL-C content was increased [38]. Lactobacillus pentosus KF923750 can reduce LDL-C and TC levels in vivo and vitro, but has no effect on HDL-C^24^. A reduction in plasma cholesterol levels was similarly observed when apoE (−/−) mice were fed 0.2 ml (1×10^9^ CFU/mL) of Lactobacillus acidophilus ATCC 4356 per day for 16 weeks under high-fat diet conditions [39]. In wild-type C57BL/6J mice fed a high-fat diet, 2 weeks of supplementation with the Lab4 probiotic consortium plus Lactobacillus plantarum CUL66 (5×10^8^ CFU/mL) resulted in significant reductions in plasma total cholesterol levels and suppression of diet-induced weight gain, but no changes in plasma levels of LDL/VLDL [40]. In contrast, in another study, the same probiotic combination and dosage not only increased plasma HDL levels but also increased total cholesterol and decreased LDL/VLDL levels in C57BL/6J mice after 12 weeks [41]. In addition to Lactobacillus, Bifidobacterium animalis VKL, and Akkermansia muciniphila also have beneficial effects such as lipid and glucose lowering, which can exert beneficial effects on diseases such as cancer and epilepsy, as well as reducing blood cholesterol and thus cardiovascular risk [42–45]. In contrast, in a clinical trial, probiotic strains of Lactobacillus acidophilus and Bifidobacterium animals, provided in either the yogurt or capsule form, did not improve cardiovascular risk factors since they did not modify the concentrations of LDL-C and HDL-C in overweight or obese individuals [46]. From the data obtained, thus far, different probiotic strains or probiotic combinations, as well as different feeding lengths, can all have an impact on the ultimate effect produced by probiotics. It is unclear whether this difference is related to the type of patients receiving probiotic treatment, the choice of probiotic strain, and the length of time that probiotics or their associated products have been ingested.

In addition, the cholesterol level in the body is usually regulated under homeostatic conditions, and excessive reduction of LDL-C will likewise bring about other health problems [47]. There are currently no studies evaluating whether this hypocholesterolaemic effect of probiotics, under long-term feeding conditions, would further reduce cholesterol levels in the body, thereby disrupting cholesterol homeostasis in vivo. Therefore, more rigorous research strategies contrasting the differences in the effects of different probiotic combinations at different feeding times should be employed in future studies.

Thus, probiotics may still be a novel pharmaceutical alternative treatment modality for patients with AS caused by cholesterol metabolism imbalance, but the specific mechanism by which probiotics regulate peripheral blood dyslipidemia remains unclear. The liver is a major organ involved in lipid metabolism, and investigators have suggested that this effect of probiotics may be associated with improved liver function, including altered expression of genes involved in liver disease, alleviation of high-fat diet-induced liver injury, and elevation of small heterodimer partner and cholesterol-7*α*-hydroxylase mRNA expression [24, 40, 41]. There is also the potential for probiotics to exert antiatherosclerotic effects by inhibiting the intestinal absorption of cholesterol, such as Lactobacillus acidophilus ATCC 435639 [39]. In addition, gut microbes combined with multiomics studies, for example, proteome and metabolome analyses, to identify the key mediators of probiotic regulation in extraintestinal organs, such as the liver, may also be helpful to elucidate the mechanisms of probiotic regulation on peripheral lipid profiles.

### 2.2. Probiotics Improve Endothelial Dysfunction

Endothelial dysfunction is a hallmark of the initiation of AS and a key event in the early stages of AS pathogenesis [48]. Endothelial dysfunction results from disrupted vascular homeostatic regulatory mechanisms. Factors contributing to the disruption of vascular homeostasis mainly include oxidative stress and inflammatory response [49]. Recently, studies have observed that probiotics may improve endothelial dysfunction by inhibiting oxidative stress and vascular inflammation, restoring endothelial structure, and increasing nitric oxide (NO) availability, potentially delaying the progression of AS at an early stage [50–52].

NO is a soluble gas with vasodilator promoting functions, as well as anti-inflammatory and antioxidant properties that can play an important role in maintaining vascular homeostasis [53, 54]. Decreased production and sensitivity of NO can lead to imbalance of vascular homeostasis and thrombosis, thereby potentially facilitating the progression of AS [55]. The utilization of probiotics to promote NO production and bioavailability may represent a novel antiatherosclerotic mechanism. Lactobacillus coryniformis CECT5711, a probiotic isolated from goat cheese, inhibits LPS and TNF-*α* expression in obese mice and reverses endothelial dysfunction in mice by increasing NO bioavailability [56]. Lactobacillus casei significantly elevated NO production in HT-29 cells (a human colon cancer cell line) [57]. But this role has not been demonstrated in vascular endothelial cells or animal model. Endothelial cells are the main source of NO, because it can constitutively express eNOS [58]. Therefore, more studies focusing on the regulation of endothelial NO production and utilization by probiotics should be performed in the future.

Lactobacillus fermentum CECT5716 can ameliorate tacrolimus induced endothelial dysfunction by inhibiting gut flora dysbiosis, downregulating NOX2 expression, and preventing eNOS uncoupling, thereby reducing vascular oxidative stress and inflammation after two weeks at a dose of 10^8^ CFU/day [26]. These findings suggest that the mechanism by which probiotics alleviate vascular endothelial dysfunction may be related to the inhibition of oxidative stress. Oxidative stress is a crucial factor leading to endothelial dysfunction, including direct damage to the vascular endothelium, induction of inflammation, and oxidation of LDL-C, inhibition of excessive oxidative stress response in vascular endothelium has been proven effective in counteracting the progression of AS [59–61]. Some probiotics have been shown to exhibit superior antioxidant capacity. Lactococcus lactis MG5125, Bifidobacterium bifidum MG731, and Bifidobacterium animalis MG741, effectively elevates total antioxidant capacity and alleviates H_2_O_2_ induced oxidative stress both in mice and HepG2 cells. The authors suggested that this effect may be related to the upregulation of glutathione and the production of several antioxidant enzymes by these probiotics. Additionally, the authors also found that these probiotics may have an important role in mediating lipid peroxidation [62]. Oxidation of LDL-C is considered to be crucial to the progression of AS [63]. Because ox-LDL is a major factor that induces endothelial cell injury, promotes macrophage foamy and atherosclerotic plaque generation [64, 65]. Study have shown that Lactobacillus plantarum NJAU-01 can alleviate oxidative stress by increasing the activity of enzymes involved in antioxidation and reducing the level of lipid oxidation in mice. The antioxidant capacity was improved with increasing concentrations of Lactobacillus plantarum NJAU-01 [66]. Study has found that Lactobacillus fermentum DR9 also decreased plasma lipid peroxidation levels in Sprague-Dawley rats [67].

Oxidative stress can also lead to damage to the structure of the vascular endothelium [68]. The changes in the structure of the vascular endothelium and endothelial barrier are directly related to the development of AS, it is possible that an impaired endothelial structure or endothelial barrier contributes to the further enlargement of atherosclerotic plaques [69]. In relative terms, the research between probiotics and vascular endothelial structure repair is still quite limited at present, but there has been related evidence showing a close relationship between them. Friques et al. found that probiotic Kefir (even at a low dose) could attenuate endothelial dysfunction of spontaneously hypertensive rats by reducing intravascular ROS production as well as restoring intravascular NO availability. Surprisingly, Kefir may also be involved in the repair of vascular endothelial structures by mediating reendothelialization of the vascular system [70]. Therefore, probiotics have the potential to be used as functional foods and therapeutic agent for the prevention of oxidative stress, to participate in the treatment or prevention of diseases such as AS.

The beneficial effects of probiotics on endothelial dysfunction have also been found in several clinical studies. In a randomized double-blind placebo-controlled 12-week trial of 81 Caucasian women, supplementation with a compound probiotic consisting of Lactobacillus, Bifidobacterium, Lactococcus, Streptococcus, and Enterococcus reduced systolic blood pressure, IL-6, TNF-*α*, thrombomodulin, and other parameters associated with endothelial dysfunction. Notably, the high dose (1×10^10^ CFU/day) of probiotics showed a more significant effect than the low dose (2.5×10^9^ CFU/day) in this study [69]. However, not all probiotics have this effect. In a study of 30 patients with metabolic syndrome treated with Lactobacillus casei Shirota (1×10^8^ CFU/day), no significant changes in relevant parameters of low-grade inflammation or endothelial dysfunction were observed after 12 weeks, although soluble vascular cell adhesion molecule 1 levels were significantly reduced [71]. Higher total amounts of probiotics may elicit more significant effects. It is unclear whether this result was related to an excessively low total amount of probiotics ingested. The main limitation of these two studies is the relatively small number of individuals examined. Therefore, further studies on the effects of probiotics on endothelial dysfunction are warranted.

### 2.3. Regulation of Inflammatory Factor Production by Probiotics

It is well-known that inflammatory response has an important role in mediating AS progression and plaque stability [72]. A key role in inflammation is played by cytokines and a variety of cytokines have been found to play key roles in the progression of AS, among which TNF-*α*, C-reactive protein (CRP), and IL-6 are secreted at all stages of AS progression and have therefore received considerable attention. In the early stages of AS, these cytokines can induce endothelial cell activation, exacerbate the production of adhesion molecules and chemokines leading to endothelial dysfunction, and subsequently induce the migration of immune cells such as monocytes into the atherosclerotic lesion site [73]. In the late stage of AS, proinflammatory cytokines can lead to atherosclerotic plaque rupture and thrombosis [74]. Therefore, anti-inflammatory therapy is a highly attractive therapeutic strategy. Several clinical trials have also achieved exciting results that modulating inflammatory status in vivo can prevent AS and its complications [75, 76].

TNF-*α* production in atherosclerotic plaques as well as increased TNF-*α* level in the blood are directly associated with AS progression and plaque size [77]. At present, some studies have found that probiotics such as Lactobacillus mucosae NK41, Bifidobacterium longum NK46, Bifidobacterium BR03 (DSM 16604), and B632 (DSM 24706) reduce TNF-*α* levels in vivo, thus potentially slowing the progression of AS [78, 79]. The regulatory effect of probiotics on the release of inflammatory factors may be associated with the expression changes of miRNAs in the gut [80]. Lactobacillus plantarum 299v (1×10^10^ CFU/day) was reported to inhibit TNF-*α* mRNA expression after 3 weeks, possibly by regulating miRNA-450a expression [81]. In contrast, there were no significant changes in TNF-*α*, IL-6 and IL-1b, and cortisol concentrations in either the Lactobacillus plantarum 299v (1×10^10^ CFU/day) or placebo groups in 60 patients with major depression after 4 weeks of intervention [82]. For both studies, the main difference was the different study subjects who received probiotic treatment. This suggested that the anti-inflammatory effects of probiotics have achieved some success in animal experiments, such as in mice and piglets. More large-scale randomized double-blind human experiments are still necessary, and the intestinal status of subjects should be carefully evaluated before administering probiotic treatment.

IL-6 is a pleiotropic cytokine that regulates the acute phase response and chronic inflammation in vivo. IL-6 is significantly higher in the peripheral blood of patients with CAD than in healthy subjects and rises progressively with the severity of the lesions; thus, it has been considered a potential biomarker to promote the progression of AS and can predict the risk of AS [83, 84]. In a study on colon cancer patients, after administration of a mixture of six probiotics, consisting of Lactobacillus acidophilus BCMC12130, Lactobacillus lactis BCMC12451, Lactobacillus casei subsp BCMC12313, Bifidobacterium longum BCMC02120, Bifidobacterium bifidum BCMC02290, and Bifidobacterium infantis BCMC02129, for 4 weeks after surgery for colon cancer, the peripheral blood proinflammatory cytokines TNF-*α*, IL-6, and IL-22 were significantly reduced [85].

CRP, an inflammatory factor mainly produced in the liver by stimuli such as IL-6, IL-1*β*, and TNF-*α* is a sensitive indicator of the inflammatory status in vivo, which can lead to vascular endothelial cell damage, participate in thrombosis, prevent the repair and proliferation of vascular endothelial cells, and thus participate in the development of AS [86, 87]. CRP is detectable early in the formation of atherosclerotic lesions and accumulates gradually with the progression of AS, so it has been studied as an independent predictor to assess the risk and progression status of AS [88]. After 8 weeks of administration of a compound probiotic capsule consisting of acidophilus (2 ×10^9^ CFU/g), Lactobacillus casei (2×10^9^ CFU/g), and Bifidobacterium bifidum (2 ×10^9^ CFU/g), subjects exhibited a significant reduction in high sensitivity C-reactive protein (hs-CRP) [89]. Similar findings have also been reported in which a complex probiotic capsule (8×10^9^ CFU/day) composed of Lactobacillus acidophilus, Bifidobacterium lactis, Lactobacillus reuteri, and Lactobacillus fermentum in combination with vitamin D administration for 12 weeks resulted in a significant decrease in hs-CRP and MDA (malondialdehyde) levels and an increase in total antioxidant capacity in the peripheral blood of women with polycystic ovarian syndrome [90]. Overall, although the current study is not sufficient to demonstrate that the direct regulation of inflammatory factor production by the gut microbiota is involved in the progression of AS, these studies provide us with new directions and targets and new ideas for the prevention and treatment of ASCVD.

### 2.4. Regulation of Macrophage Polarization by Probiotics

AS is a chronic inflammatory disease, and multiple immune cells are involved in the progression of AS. Among them, the key role of macrophages during the progression of AS has been reviewed. In brief, macrophages can play an important role in all stages of AS, and the main feature of AS is that LDL-C is taken up by macrophages and accumulated intracellularly, which eventually converts macrophages into foam cells, leading to plaque lipid storage and continued growth [91]. Macrophages can also secrete a variety of abundant proinflammatory factors, chemokines, ROS, and NO to maintain the local inflammatory response and promote further recruitment of other types of immune cells. In this process, macrophages can also interact with vascular smooth muscle cells (VSMCs) and prolong the inflammatory cycle by producing proinflammatory cytokines and extracellular matrix components, which can promote or aggravate lipid retention and plaque formation and growth [92].

With continuous research on macrophages, studies have shown that macrophages, stimulated by multiple factors in the plaque microenvironment, can exhibit different polarization states and have different functions. M1-type macrophages are characterized by proinflammatory effects and contribute to plaque growth and the transition of stable plaques to unstable plaques. M2 macrophages reduce plaque size and enhance plaque stability, playing a preventive role against the progression of AS [93]. Mhem is a novel macrophage phenotype that may exert antiatherosclerotic effects by inhibiting oxidative stress injury in plaques. In addition, M4 macrophages have dual effects on human AS, whereas MOX macrophages are proatherogenic [94]. Therefore, regulating the polarization state of macrophages has a key role in the progression of AS.

Numerous studies have shown that a variety of probiotics modulate macrophage polarization. Lactobacillus plantarum CLP-0611can induce M1 to M2 type conversion of macrophages [95]. There are also some probiotics such as Bacillus subtilis, Lactobacillus acidophilus La1 that can induce M2 macrophage polarization and decrease the proportion of M1 macrophages [96–98]. From the available evidence, few studies have focused on the polarization mechanisms of macrophages regulated by probiotics; however, it appears that probiotic-induced polarization of macrophages may be associated with amelioration of gut flora dysbiosis and metabolites. For example, gut dysbiosis promotes M2 polarization and allergic airway inflammation via fungal-induced prostaglandin E2 [99]. The gut microbial metabolite butyrate also promotes M2 macrophage polarization, both in vivo and in vitro [100]. In addition, there are studies that provide some interesting insights, such as the direct action of microorganisms on cells. Akkermansia muciniphila increases the proportion of M2/M1 macrophages in vitro, and the beneficial effects observed with administration of Akkermansia muciniphila may be mediated by one of its surface molecules, the pili-like protein Amuc_1100 [25]. There is also a view that various classes of metabolites of microbes act primarily on intestinal mucosal cells through direct contact, and how these metabolites disseminate to peripheral organs is not known. Because of the special structure of the gut, various microbes do not actually cross the intestinal epithelium into the peripheral blood, while microbial nucleic acid components detected in the plaque suggest that the interaction of gut microbes with the host may be mainly mediated by extracellular vesicles (EVs) released by microbes [101]. A recent study found that Lactobacillus plantarum-derived EVs promote human monocytic THP 1 cells to acquire an anti-inflammatory M2 phenotype, especially M2b, by inducing biased expression of cell surface markers and cytokines associated with M2 macrophages [102].

From this, it appears that probiotics have an important role in regulating the polarization of macrophages. Notably, however, most current clinical studies on probiotics employ a mixture of multiple probiotics, but the effects of microorganisms are extremely diverse and often strain specific. Although the above studies demonstrate that a mixture of certain probiotic strains can decrease the risk of AS, other studies have that some probiotic strains can also increase the production of proinflammatory cytokines and induce M1 macrophage polarization (Table 1). Therefore, the functional identification of specific probiotic strains is particularly important, which will help to clarify the role of specific probiotics in AS progression and subsequent clinical applications.

## 3. Effects of Probiotics on Novel AS Progression Factors

The association of various microorganisms with AS was progressively revealed with the development of 16S rRNA gene amplicon sequencing and shotgun metagenomic sequencing. Numerous studies have confirmed the association between compositional differences in the gut flora and AS [114–116]. In addition, the nucleic acid components of some parenteral pathogenic bacteria have been successively identified in atherosclerotic plaques, such as Porphyromonas gingivalis, Helicobacter pylori, and Chlamydia pneumoniae, which are also important for the progression of AS [117–119]. Therefore, imbalances in gut microbial homeostasis or infection with pathogenic microbes may serve as novel risk factors for AS. However, clinical anti-infective treatment did not achieve the expected effects, and may confer some additional deleterious effects [120]. Antibiotics likewise have an effect on the beneficial flora in the gut, which in turn disrupts gut flora homeostasis. Therefore, probiotics benefited from their regulatory effects on the homeostasis of intestinal flora and potential bacteriostatic effects on pathogenic microorganisms once again became the focus of AS prevention and treatment.

### 3.1. Probiotics Adjust Gut Flora Homeostasis

The gut-heart axis refers to the bidirectional relationship between the gut microbiota and the heart [121]. Recent studies have suggested that gut-heart axis imbalance resulting from an altered gut microbiota plays an important role in the progression of AS [122]. Previous studies have compared stool samples from healthy individuals as well as from patients with symptomatic AS, such as myocardial infarction and stroke, and found clear differences in the composition of the flora between the two groups of stool samples, with significant enrichment of the genus Collins in the feces of patients with symptomatic AS [123]. In addition, the abundances of gut microbes such as Bacteroides, Clostridium, and Lactobacillus have been shown to be useful in predicting coronary artery disease [8, 124]. From this, it appears that gut microbiota imbalance is one of the important factors in the development and progression of AS, and that adjusting the gut microbiota may have beneficial effects on the progression of AS, whereas probiotics seem to be an important factor in maintaining gut microbiota homeostasis.

Peng et al. found that the levels of probiotics such as Bifidobacterium and Lactobacillus in the intestine of rats with CHD were decreased, while harmful bacteria such as Actinomycetes and Desulfovibrio were significantly increased [114]. However, the application of probiotics, such as Bifidobacteria, can produce antagonistic effects on some specific harmful bacteria in the gut and can increase the levels of beneficial microorganism taxa [27]. Liang et al. found that a compound probiotic consisting of Lactobacillus and Bifidobacteria significantly altered the composition of the gut microbiota in mice fed a high-fat diet, which resulted in a significant reduction in the number of Actinobacteria and Firmicutes [125]. Similarly, in another study, a mixed probiotic consisting of Lactobacillus rhamnosus, Lactobacillus acidophilus, and Bifidobacterium breve also produced significant reduction in the abundance of Firmicutes, Actinobacteria, and Bacteroidetes in the gut of mice fed a high-fat diet [126].

In addition, as the most important digestive organs of the human body, gut microbiota imbalance can also eventually lead to a change in the human metabolic status, with an increase in harmful metabolites. There have been extensive data demonstrating that TMAO, a microbial metabolism related product, is a key pathogenic contributor to AS [127–129]. The introduction of suitable probiotic strains to modulate the microbiome suppresses TMAO production and reduces the abundance of strains involved in TMAO synthesis, thereby counteracting the progression of AS in mice [130]. This potentially beneficial effect of probiotics, while it has surprising results in mice, has not been clearly demonstrated in human clinical studies. In at least three studies showing no effect of probiotics on the content of TMAO in peripheral blood, the probiotics used included Lactobacillus casei Shirota (6.9×10^9^ CFU/day, 12 weeks); a probiotic group (1.32×10^11^ CFU/day, 4 weeks) of Lactobacillus acidophilus, Lactobacillus rhamnosus GG, Bifidobacterium animalis, and Bifidobacterium longum daily; and a probiotic group (9×10^13^ CFU/day, 4 weeks) of Streptococcus thermophilus, Lactobacillus acidophilus, and Bifidobacteria longum [131–133]. We suggest that the reason for this difference may be related to differences in the composition of the native gut flora between individuals, as rodents and humans have significantly different microbiomes [134]. In addition, alterations in gut microbiota may lead to other metabolic impairments that arise with AS, such as metabolic impairment of the S-adenosylmethionine cycle and homocysteine accumulation [135, 136]. However, there are currently no studies showing the involvement of probiotics in the production of these metabolites. Considering that there are still large gaps to be filled in regarding the relationship between probiotics and metabolic diseases, the link between probiotics and many metabolic disorder related diseases will become increasingly clear as future research continues.

The abnormal glucose metabolism is another pathogenic factor of AS in addition to lipid metabolism. Available evidence supports that blood glucose fluctuation is the etiology of lower extremity AS [137]. Although a direct causal relationship between elevated blood glucose and AS remains undefined, Tabit et al. suggested that elevated blood glucose may act with the liver or adipose tissue and through these tissues influence the progression of AS [138, 139]. Alterations in the gut microbiota also appear to be associated with abnormal glucose metabolism. A genome-wide association study showed that patients with type 2 diabetes had an altered gut flora profile, with an increase in opportunistic pathogens and a decrease in butyrate producing bacteria [140]. In a study of probiotic supplementation on insulin resistance in pregnant women with diet-controlled gestational diabetes, after taking a compound probiotics supplement consisting of Lactobacillus acidophilus (1×10^9^ CFU/day) and Bifidobacterium bifidum (1×10^9^ CFU/day) for four weeks, it significantly reduced fasting glucose and increased insulin sensitivity in women with gestational diabetes [141]. In this study, the authors did not discuss the mechanism of this probiotic's supplementation, but in another animal experiment it was shown that the glucose lowering effect of probiotics may be associated with the improvement of intestinal flora disorders. A compound probiotic derived from traditional fermented cheese whey has been shown to reduce fasting blood glucose and glycated hemoglobin in mice, which may be related to multiple probiotics by protecting islet function and modulating intestinal flora disorders [142]. However, specific probiotic strains were not published by the authors. But it is still foreseeable that the modulation of blood glucose by probiotics may have beneficial effects in patients with atherosclerosis or in those at high risk.

Tailoring gut microbiota homeostasis with probiotics or their associated products may therefore be an important means of preventing or treating AS. Currently, relevant dairy products, which include Lactobacillus and Bifidobacteria are available and have been found to be effective in reducing CVD risk in clinical studies [143]. However, it is worth noting that all types of probiotic products belong to exogenous bacteria and are not suitable for all individual applications. There is evidence that although beneficial in healthy individuals, partial probiotics elicit serious adverse effects when inoculated into the gut of individuals with disrupted gut flora balance due to disease [144]. Therefore, the application of probiotics to restore the homeostasis of the intestinal flora still needs more clinical studies to evaluate their safety, as well as more accurate assessment of the intestinal conditions of the recipients. Differences in the composition of the human microbiota resulting from habitual diet and genetic make-up, and hence differences in treatment efficacy, need to be considered more cautiously in future studies.

### 3.2. Probiotics Resist the Pathogens that Drive the Progression of AS

In addition to the gut, a variety of extraintestinal pathogenic microorganisms, such as Porphyromonas gingivalis and Helicobacter pylori, were found to accelerate the progression of AS by regulating lipid metabolism, inducing inflammation or damage to the vascular endothelium, and regulating the transformation of macrophages into foam cells [117, 145–148]. Investigators have attempted to use antibiotics against pathogenic bacteria to delay or halt the progression of AS, but clinical anti-infective therapy has not yielded good results with regard to the adverse cardiovascular events caused by AS [120, 149]. Because of the resistance of bacteria and the abuse of broad-spectrum antibiotics, the safety and efficacy of antibiotics for the treatment of AS deserve further consideration. Vancomycin has been shown to interfere with SCFAs and bile acid metabolism in vivo, thus potentially leading to a disturbance of lipid metabolism in the body and aggravating AS [150]. The development of new therapeutic modalities against AS is needed.

Probiotics have been reported to produce many metabolic byproducts such as bacteriocins, organic acids, acetaldehyde, diacetyl, ethanol, and hydrogen peroxide, which are nontoxic and nonpathogenic, and are considered the most promising alternatives to antibiotics due to their biological activity and inhibitory properties against pathogenic microorganisms of the host [151]. For example, Lactobacillus casei, Lactobacillus fermentum, Lactobacillus gasseri, Lactobacillus rhamnosus, and Lactobacillus reuteri are effective against Porphyromonas gingivalis and Helicobacter pylori, and the antibacterial effects of these probiotics probably result from competing for the substrate and binding site and producing antimicrobial compounds such as lactic acid [152–154]. Therefore, there is potential application of these probiotics, not only in the treatment of periodontitis, gastritis and other diseases but they may also have a certain preventive effect on AS. However, there is still a lack of direct evidence that probiotics slow down the development and progression of as by inhibiting potentially pathogenic bacteria associated with AS.

## 4. Conclusions

Probiotics can exert significant beneficial effects on numerous traditional as well as novel atherogenic risk factors, including gut microbiota homeostasis, cholesterol levels, proinflammatory cytokines, atherogenic associated microbes, and macrophage polarization, making probiotics a highly attractive biological therapy to prevent and treat atherosclerosis in recent years, and yielded a wide range of potential therapeutic targets.

It is important to note, however, that much of the current research on probiotics and atherosclerosis remains in correlation studies, and the mechanisms of probiotic antiatherosclerosis remain undefined, which limits the clinical use of probiotics and the development of related probiotic products, and future research directions should focus more on longitudinal or multiomics studies, thereby elucidating the function of probiotics. More importantly, the effects of microorganisms are usually strained specific, not all probiotics have antiatherosclerotic properties, part of probiotics may even have proatherosclerotic effects, which is also one of the reasons why probiotics are currently only applied as nutritional supplements rather than pharmaceuticals. Therefore, more studies are needed to clarify the mechanism of action of specific probiotic strains against atherosclerosis and to carry out large clinical studies to examine their efficacy and safety. Overall, probiotics hold promise as a new avenue for atherosclerosis prevention and treatment.

## Figures and Tables

**Table 1:** A few published studies examining the effects of probiotics on traditional atherosclerosis progression factors.

Probiotic or probiotics combination	Species	Outcomes	Prediction of the effect on atherosclerosis	References
Bifidobacterium adolescentis	Mice	↓ TNF-*α*	Prevention	[103]
Bifidobacterium breve BR03 and B632	Humans	↓ TNF-*α*	Prevention	[78]
Lactobacillus acidophilus ATCC 4356	Mice	↓ TNF-*α*, ox-LDL, TC, LDL-C ↑ IL-10	Prevention	[39, 104]
Akkermansia muciniphila	Mice	↓ TNF-*α*, MCP-1, IL-1*β*, LPS, Atherosclerotic plaques	Prevention	[105, 106]
Lactobacillus paracasei	Mice	↓ IL-1*α*, IL-1*β*, IL-2, TNF*α* ↑ IL-10, TGF*β*	Prevention	[107]
VSL#3	Mice	↓ Atherosclerotic plaques	Prevention	[108]
Lactobacillus plantarum DR7	HepG2 cell line	↓ Cholesterol	Prevention	[109]
Bifidobacterium animalis subsp. lactis and Arg YG	Humans	↑ Endothelial function	Prevention	[110]
Lactobacillus acidophilus, Lactobacillus reuteri, Lactobacillus fermentum, and Bifidobacterium bifidum	Humans	↓ TG, TC, VLDL, CRP ↑ Total antioxidant capacity, Total glutathione	Prevention	[111]
Bacillus amyloliquefaciens	Mouse BMDMs	↑ TNF-*α*, IL-6, M1 macrophages	Promoting	[112]
Lactobacillus delbruckei sp. bulgaricus DWT1 and Streptococcus thermophilus DWT4	RAW264.7 cell line	↑ IL-1*β*, IL-6, IL-12, TNF-*α*, M1 macrophages	Promoting	[113]
Lactobacillus acidophilus and Bifidobacterium animalis	Humans	No effect	No effect	[46]
